# Effects of autogenic and reciprocal inhibition techniques with conventional therapy in mechanical neck pain – a randomized control trial

**DOI:** 10.1186/s12891-022-05668-0

**Published:** 2022-07-25

**Authors:** Mahrukh Siddiqui, Saeed Akhter, Aftab Ahmed Mirza Baig

**Affiliations:** 1 Department of physiotherapy, Institute of Physical Medicine and Rehabilitation, Dow University of Health and sciences, Karachi, Pakistan; 2 Department of physiotherapy, Sindh Institute of Physical Medicine and Rehabilitation, Karachi, Pakistan

**Keywords:** Neck ache, Manual Therapies, Therapy-Soft tissue

## Abstract

**Background:**

Neck pain is a common musculoskeletal issue that has been seen as high in terms of disability. Muscle Energy Techniques (MET) are advanced soft tissue techniques to treat Mechanical Neck Pain (MNP). This study compares the Autogenic inhibition (AI) technique with the Reciprocal Inhibition (RI) technique providing conventional treatment to improve functional outcomes.

**Methods:**

A randomized control trial was conducted at Sindh Institute of Physical Medicine & Rehabilitation, Karachi, Pakistan from August 28, 2021, to December 31, 2021 among 20–50 years old patients with Moderate intensity MNP for more than 4 weeks and with limited Neck ROMs. The sample were divided randomly and allocated into two groups (groups 1 and 2). Group 1 and 2 received 12 sessions of AI and RI with Conventional therapy respectively. The randomization sheet was generated online from randomization.com for a sample size of 80 and two groups of study ‘AI’ and ‘RI’ with a ratio of 1:1 by an independent statistician. Pain (primary outcome), range of motion, and functional disability (secondary outcomes) were assessed through visual analog scale (VAS), Goniometer, and Neck disability index (NDI) at baseline, 1st, and last session respectively. Mean and standard deviation, frequency, and percentages were calculated. Chi-square test and independent t-test compare baseline characteristics. The Repeated Measure Two-Way ANOVA compared mean VAS, NDI, and ROM. The significant *P*-value was less than 0.05.

**Results:**

The mean duration of neck pain was 8 weeks. There was a more significant (*p* < 0.001) improvement in pain (ES = 0.975), disability (ES = 0.887), neck ROMs; flexion (ES = 0.975), extension (ES = 0.965), right and left lateral flexion (ES = 0.949 and 0.951), and right and left rotation (ES = 0.966 and 0.975) in the AI group than the RI group at 12th session.

**Conclusion:**

The Autogenic Inhibition-MET is more beneficial than Reciprocal Inhibition-MET in improving Pain, Range of Motion, and Functional Disability in patients with Sub-Acute and Chronic Mechanical Neck Pain. Therefore, it is a beneficial technique to add with conventional neck pain therapy to get better treatment outcomes in MNP patients.

**Trial Registration:**

Prospectively registered on ClincalTrials.Gov with ID: NCT05044078.

## Background

Neck pain is a common musculoskeletal condition ranked 4th highest in terms of disability. Out of 291 conditions, it is 21st in overall burden [1]. However, the incidence and point prevalence of neck pain were 806.6 and 3551.1 per 100,000 population, while years living with a disability is 352 per 100,000, which has been found higher among females. The prevalence of neck pain is supposed to be increased with age. The highest burden was among men aged 45–49 years and women aged 45–54 years [2].

Non-specific neck pain is pain with a postural or mechanical basis, also known as Mechanical Neck Pain (MNP) [3]. MNP can be bearable to severely excruciating that hinders regular daily activities, like the inability to dress, concentrate on work, or sleep. It may be localized to one spot or can be spread to broader regions. It can be sharp or may feel less intense, which also leads to a stiff neck and reduced range of motion [4].

Manual therapy for MNP includes Muscle energy techniques that are advanced soft tissue active stretching techniques [5]. It involves gentle contraction of muscle that helps in relaxation and lengthening of muscles that normalize ROMs. It comprises Autogenic Inhibition and Reciprocal inhibition technique. AI and RI techniques work on the principle Autogenic and Reciprocal inhibition respectively. These techniques help to improve pain, muscle tone, circulation, stretching short muscle and fascia, strengthening weak muscles, and mobilizing joints [6].

Disability from neck pain has a significant influence on employee productivity and the household economy. The financial burden of neck pain is second to low back pain in workers including increased treatment cost, pay cuts, and reimbursement [7]. The current literature available on MNP shows that despite many research studies on MNP most of them have short-term follow-up period [8–12], a small sample size [5, 9, 10, 13–15], did not provide standard treatment [8, 16, 17], used less reliable outcome measures [8, 12], done on a limited population of NP (workers, females or males only) [10, 11] or focus only on sub-scute or mostly chronic MNP patients [10, 18]. Among all those studies done on Muscle energy technique (MET) in MNP, there is only one study that has been done on both types of MET’s, but it was a short-term study that compared AI-MET and RI-MET with static stretching (SS) exercises [8]. Due to the growing need for evidence-based treatment in MNP, small sample size, the limited population of interest or specific stage of disease, less reliable outcome tools, or short follow-up periods will surely affect the generalizability and validity of these available studies results. As concluded in a systematic review (2021) by S. Sbardella there is still limited available evidence regarding MET in MNP [18]. The current study is the novel one that has addressed the above-mentioned lacking’s in the available literature by investigating the long-term effects of AI-MET against RI-MET in both sub-acute and chronic MNP patients with a statistically calculated large sample size on both genders using reliable and valid outcome tools. This study is beneficial for therapists in providing evidence-based treatment and which technique to add to their regular practices for managing MNP. Furthermore, this study adds new advances in treatment techniques available for managing mechanical neck pain in long term.

## Objective of the study

To determine the effectiveness of Autogenic Inhibition and Reciprocal inhibition techniques with conventional therapy in Mechanical neck pain to improve Pain, Range of Motion, and Functional Disability.

## Hypothesis

There is statistically significant difference in the effects of Autogenic Inhibition and Reciprocal inhibition techniques with conventional therapy in Mechanical neck pain to improve Pain, Range of Motion, and Functional Disability.

## Methodology

### Study Design and Setting

This study is a Single Blinded, two-arm, Parallel design, Randomized Control Trial, with a group allocation ratio of 1:1. This study was conducted at the Physiotherapy Department of Sindh Institute of Physical Medicine & Rehabilitation, Karachi.

### Study population

The study population included patients who were 20–50 years old [3], have Moderate intensity Mechanical Neck Pain (3.5–7.4 cm on VAS) [8] for more than 4 weeks (Sub-acute and chronic stage) [19] with limited Neck ROMs. Patients who have any history of trauma, fracture, or surgical procedure of the cervical spine [5], signs, and symptoms of cervical myelopathy and cervical radiculopathy [4], Signs of red flags or serious pathologies, such as malignancy, inflammatory or rheumatic diseases, infections, and vascular diseases such as vertebrobasilar insufficiency [4, 5], Patients suffering from any neurological conditions like Stroke, Parkinson and Multiple Sclerosis [5], Trigger point of Upper trapezius are excluded.

### Sample size estimation

The sample size of 6 was calculated by the PASS software version 11 using 2 independent sample t-test for mean, confidence interval of 99 and 80% power of the test, mean + S.D. of Cervical ROM (Extension) of AI 73.46 + 10.10^8^ and RI 66.47 + 9.89^8^ at the last treatment session, but due to low sample size, we increased the sample size to 80, means 40 patients were randomly allocated to 2 groups. Among the sample size of 40, 10 patients were included as a margin for drop-out patients.

### Study duration

The duration of the study was from August 28, 2021 to December 31, 2021.

### Data collection procedure

**Fig. 1:**
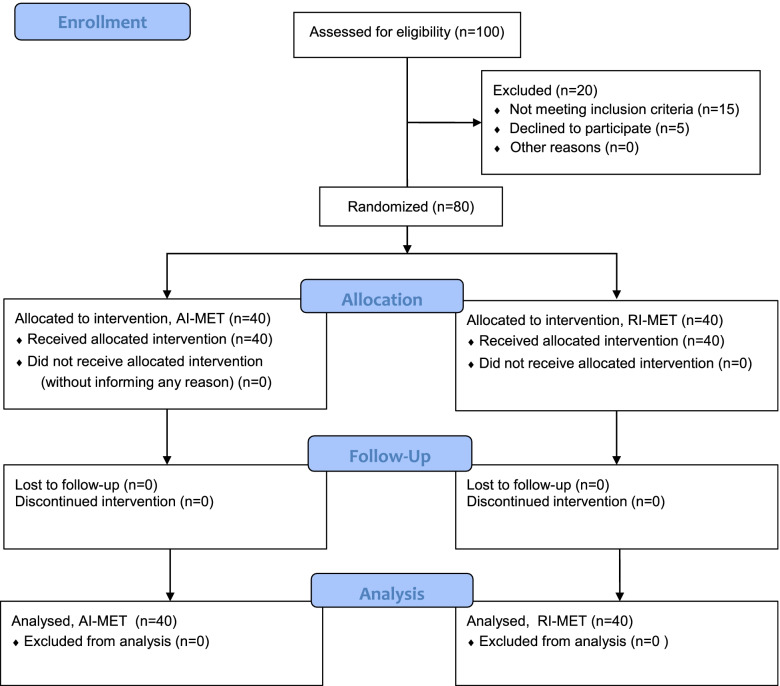
CONSORT diagram

The data collection was started after the approval from Institutional Review Board, DUHS. All the patients were recruited after diagnosis by the physiatrist, in the absence of signs and symptoms of radiculopathy and myelopathy and based on inclusion and exclusion criteria. After recruitment, informed consent was signed. After that, the patient was assigned into 2 groups, GROUP 1 (received AI with conventional treatment) and GROUP 2 (received RI with conventional physiotherapy treatment) randomly divided by computer-generated software. Total 12 sessions, 3 sessions per week for 4 weeks were provided. To study the short-term effect treatment for 2 weeks has been provided previously [5]. As our study was long-term, we have doubled the number of sessions and weeks to study the long-term effects of MET. Each session was 45 minutes long. The data was collected through Visual Analogue Scale (VAS), NDI, and goniometer before initiating the first physiotherapy session for baseline comparison of both groups and 2 minutes after the first session to get significant results about the immediate effect of MET and after the last physiotherapy session to know the long-term effects of MET. The whole procedure for assessing outcomes has taken 9–14 minutes. The CONSORT flow diagram is also given (Fig. 1).

### Consent form and questionnaire

The consent form clearly stated the study objective, any possible harms, benefits, treatment techniques of both groups, confidentiality and withdrawal information along with the consent of voluntary participation with signatures of the patients. It was available in both English and Urdu for ease of understanding. The questionnaire used in this study was Neck Disability Index which was available in both English and Urdu as well. Permission and license for each version were granted by MAPI Research to the principal investigator.

### Randomization and envelope concealment

The randomization sheet is generated online from randomization.com for a sample size of 80 and two groups of study ‘AI’ and ‘RI’ with a ratio of 1:1 by an independent statistician. Initial screening was performed based on the inclusion and exclusion criteria of the study by a consultant physiatrist having experience more than 12 years. Then patients were referred to the physiotherapy department where, after informed consent and enrolment, they were randomly allocated to one of the groups using sealed envelopes that contained the treatment according to their group.

### Masking

This study was a single-blinded randomized controlled trial in which the outcomes assessor involved in the clinical trial has been prevented from knowing the interventions assigned to individual patients.

### Intervention

All patients were given conventional therapy regardless of the study group. Conventional treatment includes Maitland PA central glides (30 oscillations, 3 sets), in Grade 1 and 2 to decrease pain on the painful segments and isometric neck strengthening exercises (10 repetitions, each 5-second hold, 1 set) followed by superficial thermotherapy provided by the hot pack for 10 minutes on the back of the neck [7].

Group 1 received AI-MET with conventional treatment while Group 2 received RI-MET with conventional treatment. AI and RI were applied to the muscles of the cervical spine, including Upper Trapezius (perform cervical extension), Sternocleidomastoid (perform cervical flexion, rotation, and lateral flexion), levator Scapulae (elevate scapula and perform cervical extension and lateral flexion), and Scalene muscles (perform cervical flexion, rotation, and lateral flexion).

AI included stretching of the affected muscle and performing isometric contraction with 50% of the total patient’s effort in the same muscle that was being stretched and position hold for 10 seconds, with 5 seconds of rest after every repetition. This procedure was repeated 5 times. The RI also included stretching of the affected muscle but contrary to AI, isometric contraction of the antagonist muscles with the 50% of total patient’s effort was followed. This position holds for 10 seconds, while agonist’s muscle was still in the stretched position, with 5 seconds of rest after every repetition. This procedure was repeated 5 times too. The only difference between both techniques is that for autogenic inhibition we performed isometric contraction of the involved muscle while for reciprocal inhibition the isometric contraction of the antagonist’s muscle was performed, the overall procedure remained the same [20].

For Upper trapezius muscle, the patient lay supine with the neck fully side bent and slightly rotated opposite from the side being treated. The therapist asked to move the ear towards the shoulder of the involved side and maintain against the resistance of the therapist’s hand for the AI technique. The therapist asked to move the ear towards the shoulder of the uninvolved side and maintain against the resistance of the therapist’s hand for the RI technique. For Levator Scapulae muscle, the patient lay supine with the neck in flexion, lateral flexion, and rotation. The therapist asked to take the head back in a neutral position towards the involved side and maintain against the resistance of the therapist’s hand for the AI technique. The therapist asked to take the head towards the uninvolved side of the chest and maintain against the resistance of the therapist’s hand for the RI technique [20].

For the Scalene muscle, the patient lay in a supine position with a folded towel under the upper back and neck in slight extension and contralateral rotation. This technique was performed in 3 positions varying in the degree of rotations to target all anterior, middle, and posterior fibers of the scalene muscle. For posterior fibers neck was in full contralateral rotation and slight extension, for the middle fiber neck was in 45-degree contralateral rotation with slight extension, and for anterior fibers neck was in less than 45 degrees of rotation with slight extension, overall procedure remained the same. The therapist asked to rotate the head towards the involved side and maintain against the resistance of the therapist’s hand for the AI technique. The Therapist asked to rotate the head towards the uninvolved side and maintain against the resistance of the therapist’s hand for the RI technique [20]. For Sternocleidomastoid muscle, the patient lay supine and the shoulders rested on a folded towel and the patient’s head in contralateral rotation away from the affected side. The therapist asked to lift the rotating head a small degree towards the ceiling and maintain against the resistance of the therapist’s hand for the AI technique. The therapist asked to lift the rotating head a small degree towards the bedside and maintain against the resistance of the therapist’s hand for the RI technique [20].

Isometric neck strengthening exercises were performed in sitting position [21] and each exercise with 10 rep and a 5-sec hold [22] was performed. The therapist told the patient to maintain the position against the therapist’s resistance. In each exercise, there was no change in muscle length. For cervical flexion, the neck was in a neutral position and the therapist asked the patient to actively flex the neck and the therapist resisted neck flexion by placing a hand on the forehead of the patient. For cervical Extension, the neck was in a neutral position and the therapist asked the patient to actively extend the neck and the therapist resisted neck extension by placing a hand on the back of the head of the patient. For cervical lateral bending on both sides, the neck was in a neutral position and the therapist asked the patient to laterally bend the neck one by one actively on both sides and the therapist resisted lateral bending by placing their hand on the side of the head of the patient. For cervical rotation on both sides, the neck in a neutral position, and the therapist asked the patient to rotate the neck one by one actively on both sides and the therapist resisted neck rotation by placing a hand on the side of the head of the patient [21].

### Outcome measures

The primary outcome measure used in this study were VAS for pain [23] (Level II evidence) [7], and secondary outcome measures are Cervical Goniometry for Range of motion [24] (Level I evidence) [7], and NDI for Functional disability [25] (Level II evidence) [7].

### Visual analogue scale

The VAS is considered to be one of the best measures of pain intensity. The VAS is a self-reported measurement consisting of a vertical line with extreme anchors of ‘no pain’ to ‘extreme pain’. This line represents a continuum of pain intensity and is 10 cm in length. The patient was asked to mark their perceived level of pain intensity (for a specified time frame) on the line. The examiner scored the instrument by measuring the distance, in cm and mm, from the ‘no pain’ anchor to the mark, which the patient identified as their level of pain. Test–retest reliability has been reported to be high for the VAS (ICC = 0.71–0.99). Concurrent validity has been found to be moderate for the VAS (0.71–0.78) [23]. The cut-off value of VAS for mild is < 3.4, moderate is 3.5–7.4 and severe is > 7.5 [26].

### Goniometer

Excellent intra-rater reliability was present with Intraclass Correlation Coefficients (ICC- 3,k) for goniometry ≥0.94 and digital inclinometry ≥0.95. The concurrent validity between goniometry and digital inclinometry was good with ICC (3,k) values of ≥0.85 [27]. The testing position was sitting, with the back supported by the chair. For reference, the patient was asked to hold a tongue depressor between their teeth. To prevent flexion of the thoracic and lumbar spine therapist stabilized the shoulder girdle. This procedure took 5 minutes to complete [24].

For Cervical Flexion and Extension, the goniometer center was placed over the center of the ear. The proximal arm was placed perpendicular or parallel to the ground. The distal arm was moved with nose tip or parallel to the longitudinal axis of the tongue depressor. For Cervical Lateral Flexion both sides, the goniometer center was placed over the C7 spinous process. The proximal arm was placed perpendicular to the ground. The distal arm was moved with the midline of the back of the head. For Cervical Rotation both sides, the goniometer center was placed on top of the head. The proximal arm was placed in parallel to the imaginary line between 2 acromial processes. The distal arm was moved with the nose tip parallel to the longitudinal axis [24].

### Neck disability index (NDI)

The NDI is available in English and Urdu. The NDI has been found to possess excellent test-retest reliability, strong construct validity, strong internal consistency, and good responsiveness in assessing disability in patients with mechanical neck pain (MNP) [28]. The NDI (Urdu) is also a reliable and valid tool to measure disability in Urdu-speaking patients with MNP [29]. Permission for both English and Urdu versions was obtained from MAPI Research trust. It has 10 sections which are scored 0 to 5, in which 0 means ‘No pain’ and 5 means ‘Worst imaginable pain. The patient rated all 10 items including pain, personal care, lifting, reading, headaches, concentration, work, driving, sleeping, and recreation. Points were summed to get a total score. After the total sum of the score, 0–4 is no disability, 5–14 means mild disability, 15–24 means moderate disability, 25–34 means severe disability, and 35–50 is a complete disability. About 3 to 8 minutes was taken to complete this questionnaire [30].

### Harms and adverse events

There are no harms and adverse event reported during the period of trial.

### Data analysis procedure

The Statistical Package of Social Sciences was used to analyze all the data. Mean and standard deviation calculated for Continuous variables. Frequency and percentage calculated for categorical variables. The association of the demographic variables between both groups at baseline is shown through the Chi-square test. An independent t-test was used to compare baseline characteristics of continuous variables. The Shapiro Wilk test statistically checked the assumptions of normality. For means comparison of VAS, NDI, and ROM, Repeated Measure Two-Way ANOVA was applied. *P*-value < 0.05 was considered significant. The result is presented in the form of tables. For categorical variables, frequency and percentages were shown for each group separately and combined. Educational and occupational status are presented in a pie-chart while the stage of the disease is shown by a bar chart. Also, for continuous variables mean, standard deviations, and lower or upper limit were shown for each group separately and combined. In another table, we present mean, standard deviation, lower and upper limit of VAS, NDI, and ROMs for baseline, 1st session, and the last session separately for both groups. Mean difference was calculated for within-group comparison. And *p*-value was shown for between-group comparisons.

## Results

The socio-demographical characteristics of all 80 patients are in Tables 1. The mean age of patients was 33.56 ± 9.04. The frequency and percentage of female patients were higher than the male patients. Among them, more than half of the study sample had married [53(66.2%)] and normal weight [53(66.2%)] (Table 1).

**Table 1:** Socio-demographical characteristics of study patients (*n* = 80)

Characteristics^a^	Group 1(AI) *n* = 40^a^	Group 2(RI) *n* = 40^a^	Total *n* = 80	*p*-value*
**Age (years)**	33.82 ± 9.31	33.30 ± 8.76	33.56 ± 9.04	0.79
**Height (meters)**	1.61 ± 0.09	1.64 ± 0.13	1.62 ± 0.11	0.24
**Weight (kg)**	60.32 ± 9.74	61.72 ± 10.63	61.03 ± 10.18	0.54
**BMI (kg/m**^**2**^**)**	23.24 ± 3.31	23.12 ± 4.34	23.18 ± 3.82	0.89
**Gender:**				
**Male**	11 (47.82%)	12 (52.17%)	23 (28.8%)	
**Female**	29 (50.87%)	28 (49.12%)	57 (71.2%)	
**Marital Status:**				
**Single**	12 (48%)	13 (52%)	25 (31.2%)	
**Married**	28 (52.83%)	25 (47.17%)	53 (66.2%)	
**Divorced**	0 (0%)	2 (100%)	2 (2.5%)	
**Stage of Disease:**				
**Sub-Acute**	19 (47.5%)	21 (52.5%)	40 (50%)	
**Chronic**	15 (37.5%)	25 (62.5%)	40 (50%)	
**Occupational Status:**				
**Unemployed/house wife**	23 (57.5%)	17 (42.5%)	40 (50%)	
**Private job**	6 (31.58%)	13 (68.42%)	19 (23.8%)	
**Government Job**	10 (50%)	10 (50%)	20 (25%)	
**Disable**	1 (100%)	0 (0%)	1 (1.2%)	
**Body Mass Index Groups:**				
**Under weight**	3 (50%)	3 (50%)	6 (7.5%)	
**Normal weight**	27 (50.94%)	26 (49.06%)	53 (66.2%)	
**Overweight**	8 (50%)	8 (50%)	16 (20%)	
**Obese**	2 (40%)	3 (60%)	5 (6.2%)	

^**a**^Values represented as mean and standard deviation or frequency (percentage); *Values presented as Level of significance with independent T test.

In Table 2, Group 1(AI) shows more significant (< 0.001) improvement in pain level as compared to Group 2(RI) at 1st and last session. The VAS scores improved more about 2.07 ± 0.72 in the AI group than the RI group, which improved to 1.94 ± 0.83. However, between groups comparison showed almost similar improvement in VAS scores in both 1st and last session as compared to baseline. The effect size of 0.975 shows large improvement in AI group as compared to RI group (Table 2).

**Table 2:** Between-group and within group comparisons of VAS (*N* = 80)

Groups *n* = 80	Baseline^a^	1st session^a^	12th session^a^	Within Group Comparison^b^		Effect Size (Between Group at 12th session)
				Baseline v/s 1st session	Baseline v/s last session	
Group 1 (*n* = 40)	5.78 ± 0.67 (5.56–6.01)	4.14 ± 0.7 (3.85–4.44)	2.07 ± 0.72 (1.82–2.31)	1.64 (< 0.001)	3.71 (< 0.001)	
Group 2 (*n* = 40)	5.63 ± 0.76 (5.56–5.86)	4.06 ± 1.12 (3.77–4.35)	1.94 ± 0.83 (1.69–2.19)	1.57 (< 0.001)	3.69 (< 0.001)	0.975
Between Group Comparison^b^ (*p*-value)	0.15 (< 0.001)	0.08 (< 0.001)	0.13 (< 0.001)			

^**a**^Values presented as mean **±** standard deviation (95% CI); ^b^Values presented as mean difference

In Table 3, Group 1 (AI) shows more improvement in disability scores at both 1st and last session as compared to Group 2 (RI). Mean disability scores for the AI group were 4.30 ± 1.87 at the final session compared to the RI group, 4.80 ± 1.84. Between groups comparison showed almost similar improvement in disability score in both 1st and last session, compared to baseline. Also, the *p*-value was < 0.05, which means a statistically significant (< 0.001) difference in disability scores between both groups present. The effect size of 0.887 shows large improvement in AI group as compared to RI group (Table 3).

**Table 3:** Between-group and within group comparisons of NDI (*N* = 80)

Groups (*n* = 80)	Baseline^a^	1st session^a^	12th session^a^	Within Group Comparison^b^		Effect Size (Between Group at 12th session)
				Baseline v/s 1st session	Baseline v/s last session	
Group 1 (*n* = 40)	22.7 ± 8.6 (20.07–25.3)	20.73 ± 8.48 (18.16–25.3)	4.3 ± 1.87 (3.72–4.89)	1.97 (< 0.001)	18.4 (< 0.001)	
Group 2 (*n* = 40)	22.75 ± 8.09 (20.12–25.4)	21 ± 7.81 (18.43–23.6)	4.80 ± 1.84 (4.22–5.39)	0.75 (< 0.001)	17.95 (< 0.001)	0.887
Between Group Comparison ^b^ (*p*-value)	−0.05 (< 0.001)	−0.27 (< 0.001)	−0.5 (< 0.001)			

^**a**^Values presented as mean **±** standard deviation **(95% CI)**; ^b^Values presented as mean difference

In Table 4, All neck ROMs improved significantly (< 0.001) in group 1(AI) as compared to group 2(RI) in both 1st and the last session from baseline. There was more substantial improvement than baseline in flexion, right lateral flexion, and right rotation ranges after 1st session and a steady improvement till the last session in both groups. There was slight improvement at both 1st and the last session in both groups for extension; left lateral flexion; left rotation. The effect size of Flexion is 0.975, extension is 0.965, right lateral flexion is 0.949, left lateral flexion is 0.951, right rotation is 0.966 and left rotation is 0.975, which shows large effect of treatment n AI group as compared to RI group (Table 4).

**Table 4:** Between-group and withing group comparisons of neck ROMs(*N* = 80)

Groups *n* = 80	Baseline^a^	1st session^a^	12th session^a^	Within Group Comparison^b^ (*p*-value)		Effect Size (Between Group at 12th session)
				Baseline v/s 1st session	Baseline v/s last session	
**Flexion:**						
**AI group**	44.82 ± 13.99 (40.89–48.76)	56.78 ± 12.91 (52.87–60.66)	75.25 ± 9.78 (72.23–78.26)	−11.96 (< 0.001)	− 30.43 (< 0.001)	
**RI group**	42.77 ± 11.91 (38.83–46.71)	53.35 ± 11.77 (49.46–57.24)	69.9 ± 9.35 (66.89–72.91)	−10.58 (< 0.001)	−27.13 (< 0.001)	0.975
**Between Group Comparison**^**b**^ **(p-value)**	2.05(< 0.001)	3.43(< 0.001)	5.35(< 0.001)			
**Extension:**						
**AI group**	30.15 ± 10.91 (26.57–33.73)	39.9 ± 10.47 (36.44–43.36)	56.3 ± 9.17 (53.51–59.09)	−9.75 (< 0.001)	−26.15 (< 0.001)	
**RI group**	32.75 ± 11.79 (29.17–36.33)	39.1 ± 11.48 (35.64–42.56)	54.82 ± 8.57 (52.03–57.62)	−6.35 (< 0.001)	−22.07 (< 0.001)	0.965
**Between Group Comparison**^**b**^ **(p-value)**	−2.63(< 0.001)	0.8(< 0.001)	1.48(< 0.001)			
**Right Lateral Flexion:**						
**AI group**	36.22 ± 14.41 (31.84–40.61)	48.18 ± 14.3 (43.88–52.47)	59 ± 9.51 (56.05–61.94)	−11.96 (< 0.001)	−22.78 (< 0.001)	
**RI group**	34.68 ± 13.42 (30.29–39.06)	42.40 ± 12.96 (38.10–46.69)	56.27 ± 9.2 (53.33–59.22)	−7.72 (< 0.001)	−21.59 (< 0.001)	0.949
**Between Group Comparison**^**b**^ **(p-value)**	1.54(< 0.001)	5.78(< 0.001)	2.73(< 0.001)			
**Left Lateral Flexion:**						
**AI group**	32.75 ± 15.06 (28.53–36.97)	41.82 ± 13.96 (37.91–45.74)	57.17 ± 6.19 (55.19–59.16)	−9.07 (< 0.001)	−24.42 (< 0.001)	
**RI group**	33.25 ± 11.54 (29.03–37.47)	39.63 ± 10.74 (35.70–43.54)	52.45 ± 6.4 (50.47–54.43)	−6.38 (< 0.001)	−19.2 (< 0.001)	0.951
**Between Group Comparison**^**b**^ **(p-value)**	−0.5(< 0.001)	2.19(< 0.001)	4.72(< 0.001)			
**Right Rotation:**						
**AI group**	48.55 ± 17.97 (42.76–54.34)	56.62 ± 15.64 (51.49–61.76)	79.92 ± 4.33 (78.34–81.50)	−8.07 (< 0.001)	−31.37 (< 0.001)	
**RI group**	54.2 ± 18.83 (48.40–59.99)	60.4 ± 16.93 (55.27–65.53)	78.32 ± 5.64 (76.74–79.91)	−6.2 (< 0.001)	−24.12 (< 0.001)	0.966
**Between Group Comparison**^**b**^ **(p-value)**	−5.65(< 0.001)	−3.78(< 0.001)	1.6(< 0.001)			
**Left Rotation:**						
**AI group**	45.22 ± 15.46 (40.54–49.90)	61.02 ± 17.5 (55.56–66.49)	85.23 ± 2.28 (84.62–85.83)	−15.80 (< 0.001)	−40.01 (< 0.001)	
**RI group**	46.40 ± 14.26 (41.72–51.08)	61.35 ± 17.21 (55.88–66.81)	84.97 ± 1.51 (84.37–85.58)	−14.95 (< 0.001)	−38.57 (< 0.001)	0.975
**Between Group Comparison** ^**b**^ **(p-value)**	−1.18(< 0.001)	−0.33(< 0.001)	0.26(< 0.001)			

^**a**^Values presented as mean **±** standard deviation (95% CI); ^b^Values presented as mean difference

## Discussion

The current study focuses on the effect of two types of METs in MNP. The study aimed to compare the effects of AI and RI with conventional therapy to improve pain, ROM, and functional disability in MNP. The current study found a significant improvement in both groups, as AI is more effective than RI with conventional therapy. These findings are by the literature that faster relaxation by autogenic inhibition pathway produces more inhibition than reciprocal inhibition pathways [31].

The current study has more patients in the age group 20-30y followed by 41-50y then 30-40y. The increased neck pain in age 20-30y might be due to COVID-19 restrictions that caused young people to limit and reduce their daily physical activities leading to musculoskeletal pain like MNP [32].

Moreover, the current study had more married patients with neck pain. It may be that married women and men are more involved in strenuous or sustained physical activities than others. However, widowed/separated are suggested to be more prone to neck pain with disturbed psychosocial status [33].

The current study also has more patients with neck pain who have higher educational status. It might be due to poor posture adapted during educational and academic activities [34]. The current study had individuals with more normal weight followed by overweight, underweight and obese. Contrary to this, another review suggested high BMI (> 30 kg/m2) as a risk factor for neck pain [35]. Inconsistent results might be due to differences in age groups and methods evaluating BMI. In the current study, half of the patients were housewives. The household works and activities mentioned before were the cause of more patients of neck pain in this group [36].

The pain intensity can be best self-reported through the VAS-10 cm scale [23]. In the current study, both groups showed significant improvement in pain level. AI group showed more improved pain in 1st and last session as compared to the RI group. With regards to immediate effects (1st session), our results are the following. The current study follows the results of M. Osama et al. in regard to the 1st session but contradicts the last session (12th session) [8]. The study conducted by M. Osama et al. showed no significant difference in pain level between both groups which is contrary to the result of the current study [8]. Also, in the current study the VAS scores reduced more after 1st session as compared last session in both groups. In contrast, the previous study by M. Osama et al. showed more improvement in pain scores at the last session as compared to 1st session in both groups [8]. This might be due to the difference between outcome measures. They used numerical pain rating scale [8] while the current study used VAS, which is more sensitive to measuring pain level [23]. Another reason could be the difference between treatment sessions. The previous study provided 5 consecutive sessions [8] in one week while the present study has provided 12 sessions over one month. Another study conducted by Sharmila on the effect of post-isometric relaxation (AI) versus conventional exercises in school teachers [37] showed similar results as the current study in reducing pain level. They used VAS to measure pain level, but patients are school teachers only and no reciprocal inhibition group [37]. Another study by Phadke et al. conducted on the effect of post-isometric relaxation (AI) versus SS also showed similar results as the current study in terms of reducing pain level [5]. They also measured pain level using VAS same as the current study, but they have no reciprocal inhibition group, and they had not measured cervical ROMs [5]. The MCID scores of VAS is suggested as 30 mm [38]. The current study achieved MCID score of 1.64 cm in group 1 and 1.57 cm in group 2 in both groups which was more than the previous studies.

Functional disability is best measured by the Neck disability index. NDI is a reliable and valid tool to measure disability in neck pain patients [28, 29]. The current study has more improvement in the AI group as compared to the RI group at the last session but similar improvement after 1st session. In contrast, a study conducted by M. Osama et al. on the effect of AI and RI with SS showed no significant difference in NDI scores between both groups at the last session [8]. This might be due to the difference in treatment sessions provided in both studies. However, another study conducted by Phadke et al. on the effect of post-isometric relaxation (AI) versus SS shows a similar result as our study at the last session [5]. Though, there was no RI group in Phadke et al.’s study and effects on cervical ROM were not observed either [5]. On the contrary, an RCT demonstrated no significant difference in disability scores between PIR, which is a type of AI, and SS on neck pain and disability in patients with cervical spondylosis [39]. Although they used NDI like the current study but the earlier study lacked an RI group and patients were patients with cervical spondylosis [39]. The current study showed more change in MCID scores, 18.4 in AI group and 17.95 RI group than suggested by Brian A Young et al. which was 7.5 [25].

The range of motion can be easily measured by a Universal goniometer. The goniometer is a reliable and valid tool to measure cervical ROM [40]. Goniometer can be used for outcome analysis after the intervention is applied to compare the effectiveness of different treatment techniques [41]. In the current study, the AI group showed more substantial improvement in flexion, right lateral flexion, and right rotation ranges after 1st session and a steady improvement till the last session as compared to baseline in both groups. For extension, left lateral flexion, and left rotation slight improvement at both 1st and the last session was found in both groups. In comparison, the previous study by Osama et al. showed greater improvement in all Neck ROMs except extension in the AI group as compared to the RI group after 1st and last session [8]. They have used the same outcome measure for ROM, but they had provided fewer sessions of the treatment (5 sessions) [8] than the current study. On the other hand, contrary to our results, a study demonstrated no significant difference (*p* > 0.05) between PIR and SS in terms of ROM in patients with cervical spondylosis [39]. They have used the same outcome measure but have no reciprocal inhibition group and patients are chronic cervical spondylosis patients [39]. According to Jorgensen R et.al., The MCID scores for Flexion 6, extension 4, Right Rotation 10, left rotation 5, right lateral flexion 5 and left lateral flexion is 5 respectively [42]. While current study has more change in MCID score in all ROMs except right rotation for both groups.

## Limitations

The findings of this study have to be seen in the light of the following major limitations. This study recruited the patients with non-probability purposive sampling technique due to the nature of patients’ characteristics requiring rehabilitation. There was comparative group instead of control due to objective of the study. However the control of all confounding factors and biasness was kept according to the consort guideline and we followed all consort guideline in the current study The change in pain intensity was limited to subjective findings of the visual analog scale only. The patients could misunderstand the requirement to complete its assessment. All patients were taught extensively regarding VAS before its administration. The ROMs measurement was assessed with a goniometer which could cause the manual error however the average value was taken to overcome this limitation. The disability measurement was limited to NDI which identifies superior responsiveness. Both English and Urdu versions were used but they lacked the emotional, social, and psychological factors related to disability. The same physiotherapist performed the intervention for both groups but was expert in both interventions equally.

## Conclusion

The present study concluded that Autogenic Inhibition (MET) is more beneficial than Reciprocal Inhibition (MET) in improving Pain, Range of Motion, and Functional Disability in patients with Sub-Acute and Chronic MNP. AI-MET showed remarkable improvement in terms of immediate and long-term effects on MNP across all outcome measures. Therefore, it is a good technique to add with conventional neck pain therapy to get better treatment outcomes in MNP patients.

## Data Availability

The data is available from corresponding author on reasonable request.

## Abbreviations

MNP: Mechanical Neck Pain
ROM: Range of Motion
AI: Autogenic Inhibition
RI: Reciprocal Inhibition
NDI: Neck Disability Index
MET: Muscle Energy Technique
VAS: Visual Analogue Scale
SS: Static Stretching
